# Contrast-Enhanced Magnetic Resonance Imaging (MRI) as a Prognosticator for the Conservative Treatment of Lumbar Disc Prolapse: A Prospective Observational Study

**DOI:** 10.7759/cureus.93249

**Published:** 2025-09-26

**Authors:** Bharat R Dave, Sandesh Subhash Agrawal, Mahesh Sagar, Shivanand C Mayi, Ajay Krishnan, Ravi Ranjan Rai, Mirant B Dave, Mikeson Panthackel, Amritesh Singh

**Affiliations:** 1 Spine Surgery, Stavya Spine Hospital and Research Institute, Ahmedabad, IND; 2 Spine Surgery, Bhavnagar Institute of Medical Sciences, Bhavnagar, IND

**Keywords:** contrast-enhanced mri, diagnostic, enhancement, lumbar disc prolapse, prospective

## Abstract

Introduction: Lumbar disc herniation (LDH) is a common spinal pathology, often managed conservatively. However, predicting which patients will show spontaneous resolution versus persistent symptoms remains a clinical challenge. This study aims to evaluate the role of contrast-enhanced magnetic resonance imaging (MRI) in predicting the natural history of disc prolapse and correlating it with clinical improvement.

Materials and methods: A prospective observational study was conducted involving 50 patients between the ages of 18 and 65 years who have been undergoing treatment for the primary diagnosis of LDH. Patients were enrolled if they have partial relief of symptoms at the end of 6-8 weeks of conservative trial. All participants underwent contrast-enhanced MRI to evaluate the presence of rim enhancement. A clinico-radiological correlation was performed based on whether or not rim enhancement was observed on the contrast-enhanced MRI.

Results: Among the 44 patients who had contrast enhancement on MRI, 42 patients (95.5%) showed a good clinical outcome at the time of final follow-up as measured by the visual analog scale (VAS) score. Among the total group, six patients (12%) required a surgical procedure for relief of their symptoms, and the majority of these patients, four out of six (66.7%), were those who had failed conservative management without any contrast enhancement on MRI.

Conclusion: Contrast-enhanced MRI can serve as a powerful diagnostic adjunct in patients with incomplete symptom resolution during the conservative management of LDH. The presence of peripheral rim enhancement may predict favorable spontaneous resolution, enabling clinicians to make informed decisions regarding continued non-operative treatment versus surgical referral.

## Introduction

Lumbar disc herniation (LDH) is one of the most common causes of lower back and radicular leg pain in adults. Conservative management remains the cornerstone of treatment, often yielding satisfactory outcomes over time [1]. However, in patients who experience only partial relief after an initial course of conservative therapy, clinicians often face a dilemma: whether to continue non-operative treatment or consider alternative modalities such as epidural steroid injections or surgical intervention. A substantial body of evidence suggests that the natural course of LDH is frequently benign, with many cases demonstrating the spontaneous regression of the herniated disc material [2-5]. The proposed mechanism behind this spontaneous resorption involves an inflammatory response at the periphery of the herniated fragment, characterized by macrophage infiltration and neovascularization [6,7].

Contrast-enhanced magnetic resonance imaging (MRI) has emerged as a non-invasive modality capable of identifying these inflammatory and vascular changes. Specifically, peripheral rim enhancement observed on contrast-enhanced MRI is believed to represent neovascularization and macrophage activity surrounding the herniated disc, indicative of an active resorption process [8-10]. Despite its theoretical utility, the clinical significance of contrast-enhanced MRI in predicting symptom resolution in LDH remains underexplored. This prospective study aims to assess how contrast-enhanced MRI can predict the natural course of LDH and its association with clinical improvement in patients managed conservatively. Our research specifically focuses on an Indian patient cohort undergoing conservative treatment, offering population-specific insights that are currently underrepresented in existing literature.

## Materials and methods

This was a single-center, prospective, observational study conducted at the Stavya Spine Hospital and Research Institute in Ahmedabad, India, from September 2022 to September 2024. Institutional ethics approval was obtained from the Stavya Spine Hospital and Research Institute Institutional Ethics Committee (approval number: SSHRI/NS/CMRI/BRD/07/092012; date: September 7, 2012). A total of 50 patients between the ages of 18 and 65 years who have been undergoing treatment for the primary diagnosis of LDH were enrolled if they have partial relief of symptoms at the end of 6-8 weeks of conservative trial. Clinical assessment of pain severity was done using the visual analog scale (VAS) score [11]. Patients with a VAS score between 4/10 and 6/10 (leg pain) at the end of 6-8 weeks were included. Those with no/complete relief of symptoms, major neuro-deficit, cauda equina syndrome, and lumbar instability or who underwent surgery before 6-8 weeks were excluded. The contained non-focal disc was excluded as well. All the enrolled group of patients had contrast-enhanced MRI in the supine position.

MRI protocol

Prior to undergoing contrast-enhanced MRI, all patients were screened with serum creatinine and estimated glomerular filtration rate (eGFR) assessments to evaluate renal function. A 1.5T MRI scanner (Philips Multiva, Netherlands) was used for imaging. Imaging sequences were T2W sagittal (time of repetition (TR)/echo time (TE) 4000/100 milliseconds, slice 4 mm, inter-slice gap 0.4 mm), T1W sagittal (TR/TE 600/08 milliseconds, slice 4 mm, inter-slice gap 0.4 mm), and T1W fat-saturated trans-axial (TR/TE 600/10 milliseconds, slice 3 mm, inter-slice gap 0.3 mm) following 15-ml intravenous gadopentetate dimeglumine (Magnilek; JB Chemicals & Pharmaceuticals Ltd, Bharuch, India). The sagittal images were acquired with a matrix of 432 and three excitations, while the trans-axial images were acquired with a matrix of 432 and two excitations. A field of view of 20-20 cm was used in axial and 18-31 cm in sagittal images. Imaging data were stored on optical discs, and analysis was performed on a workstation (iMac 21; OsiriX MD License Systems, Apple Inc., Cupertino, CA, USA). Statistical analysis was conducted using paired t-tests and descriptive statistics. A p-value of <0.05 was considered statistically significant.

MRI analysis and measurement protocol

**Figure 1:**
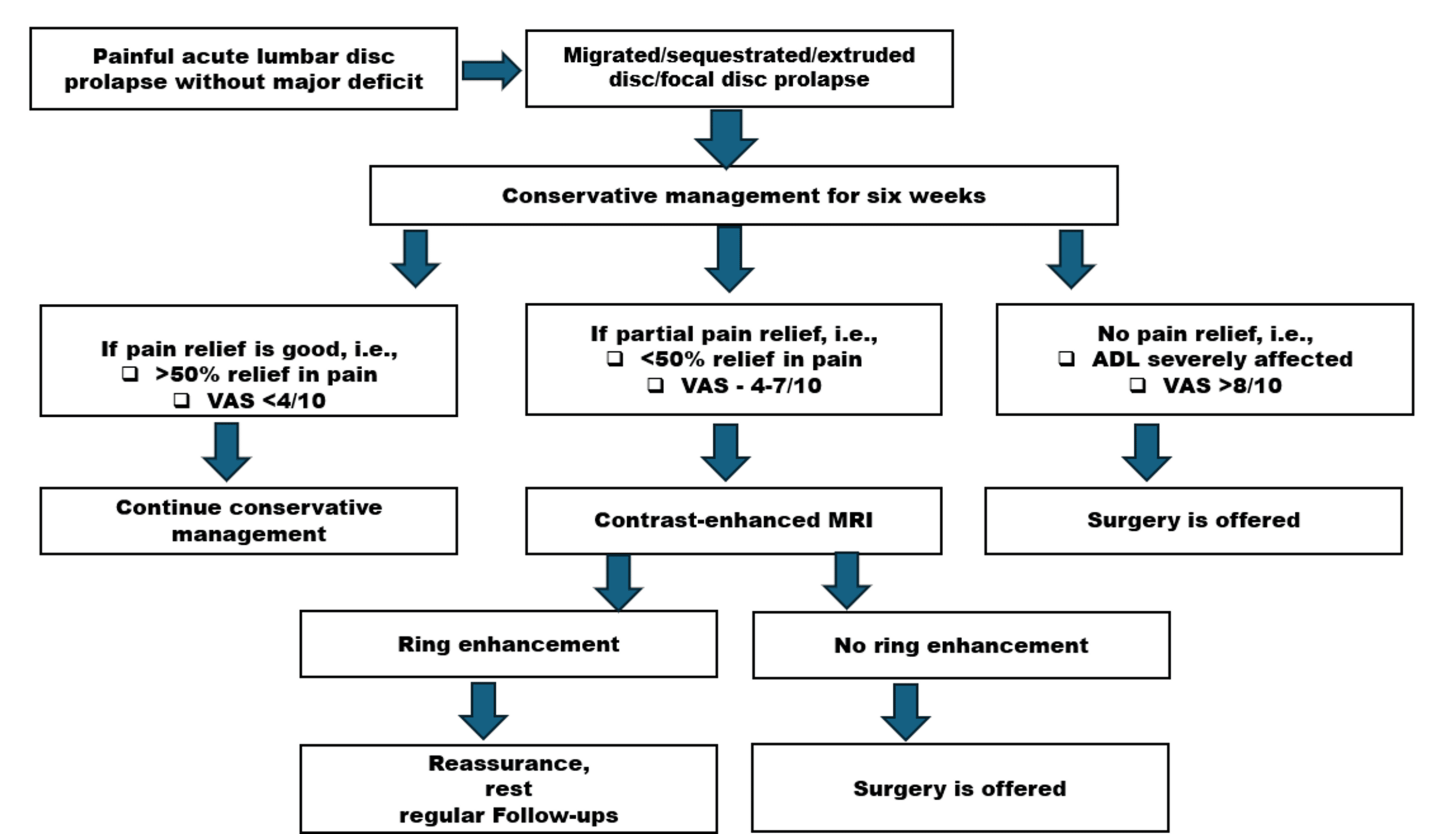
Algorithm of painful acute lumbar disc prolapse VAS: visual analog scale; ADL: activities of daily living; MRI: magnetic resonance imaging

The affected disc level was identified and categorized based on MRI morphology into protrusion, extrusion, or sequestration. The thickness of contrast enhancement, a marker of neovascularization surrounding the herniated disc fragment, was measured in millimeters on both sagittal and axial cuts at the pathological level (Figure 1). To improve measurement reliability, assessments were performed independently by two experienced radiologists who were blinded to clinical data. Inter-rater reliability was calculated using intraclass correlation coefficients (ICC), which demonstrated excellent agreement (ICC >0.85). The average of both observers' measurements in sagittal and axial planes was used for statistical analysis.

Clinical management and surgical decision-making

Patients demonstrating rim enhancement on MRI continued conservative treatment, while those without enhancement were advised surgical intervention in cases of persistent or worsening symptoms. However, surgical decision-making was not based solely on MRI findings; it also incorporated comprehensive clinical evaluation, symptom severity, neurologic examination, and patient preference after informed discussion regarding risks and benefits. VAS scores for back and leg pain were recorded at follow-up visits to monitor symptom progression, and outcomes were correlated with the presence or absence of neovascularization on contrast-enhanced MRI. The management algorithm utilized in the study is depicted in Figure 1.

## Results

A total of 50 patients were enrolled in the study, comprising 31 males (62%) and 19 females (38%), with a mean age of 36.3 ± 11.89 years (range: 18-65 years). The most commonly affected intervertebral levels were L5-S1 in 26 patients (52%) and L4-L5 in 24 patients (48%). Based on MRI morphology, 10 patients (20%) had disc protrusion, 35 patients (70%) had extrusion, and five patients (10%) had sequestrated discs. These baseline characteristics are summarized in Table 1.

**Table 1:** Baseline demographic and clinical characteristics of the study population (n = 50) Values are represented as the number of patients (n) and percentages (%), unless otherwise specified. Age is presented as mean ± standard deviation (SD). MRI: magnetic resonance imaging

Variable	Value
Age (mean ± SD, range)	36.3 ± 11.89 years (18-65)
Gender	31 males (62%), 19 females (38%)
Most common disc level	L5-S1: 26 patients (52%), L4-L5: 24 patients (48%)
MRI disc morphology	Protrusion: 10 (20%), extrusion: 35 (70%), sequestration: 5 (10%)
Patients showing rim enhancement	44 (88%)
Patients without rim enhancement	6 (12%)

Contrast-enhanced MRI revealed rim enhancement around the herniated disc in 44 of 50 patients (88%). Of the six patients without contrast enhancement (12%), four patients (66.7%) ultimately required surgical intervention due to persistent or worsening symptoms. In contrast, two of the 44 patients (4.5%) with rim enhancement needed surgery. The correlation between rim enhancement and surgical need is shown in Table 2.

**Table 2:** Correlation between contrast enhancement and surgical intervention MRI: magnetic resonance imaging

MRI finding	Total patients (n)	Surgical patients (n (%))
Rim enhancement present	44	2 (4.5%)
Rim enhancement absent	6	4 (66.7%)

The thickness of the contrast rim enhancement ranged from 1 mm to 3.8 mm, with an overall mean of 1.57 ± 0.83 mm. The average thickness was 1.55 ± 1.02 mm at L4-L5 and 1.60 ± 0.65 mm at L5-S1, as outlined in Table 3.

**Table 3:** Contrast rim enhancement thickness by disc level (in mm)

Disc level	Mean enhancement thickness (mm) ± SD
L4-L5	1.55 ± 1.02
L5-S1	1.60 ± 0.65
Overall mean	1.57 ± 0.83

At presentation, the mean VAS score was 8.64 ± 1.73, which reduced to 6.64 ± 1.58 after 4-6 weeks of conservative treatment. At the final follow-up, the mean VAS score improved further to 4.28 ± 1.67, indicating substantial symptomatic relief. Subgroup analysis revealed that the VAS reduction was statistically significant in patients with disc extrusion (p < 0.001) and sequestration (p = 0.004), while it was not significant in cases of protrusion (p = 0.109). These results are summarized in Table 4.

**Table 4:** VAS score improvement by disc morphology Values are presented as mean ± standard deviation (SD). Statistical comparisons were performed using paired t-tests. The test statistic (t-value) and corresponding p-value are shown. A p-value of <0.05 was considered statistically significant. VAS: visual analog scale

Disc morphology	Initial VAS	VAS at 4-6 weeks	Final VAS	P-value
Protrusion (n = 10)	8.3 ± 1.6	6.8 ± 1.4	5.2 ± 1.3	0.109
Extrusion (n = 35)	8.7 ± 1.7	6.6 ± 1.6	4.1 ± 1.6	<0.001
Sequestration (n = 5)	9.0 ± 2.0	6.2 ± 1.8	3.8 ± 1.2	0.004
Total (n = 50)	8.64 ± 1.73	6.64 ± 1.58	4.28 ± 1.67	-

Overall, 42 of 50 patients (84%) showed good clinical outcomes with conservative management. Among the 44 patients with rim enhancement, 42 (95.5%) improved without requiring surgery. Of the six patients who underwent surgery, four patients (66.7%) belonged to the non-enhancing group. All patients, regardless of treatment modality, had favorable outcomes at the final follow-up. A consolidated overview is provided in Table 5.

**Table 5:** Summary of clinical outcomes at the final follow-up

Outcome measure	Patients (n (%))
Good outcome with conservative management	42 (84%)
Surgery required	6 (12%)
Good outcome after surgery	6 (100%)
Rim enhancement positive with good outcome	42 (95.5% of 44)
Rim enhancement negative requiring surgery	4 (66.7% of 6)

**Figure 2:**
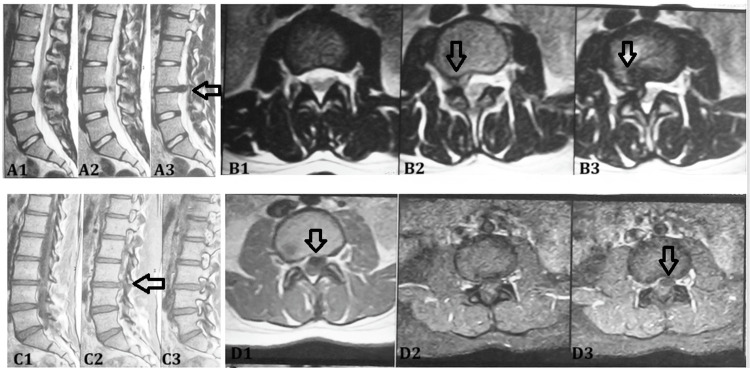
Sagittal (A1-A3) and axial (B1-B3) T2-weighted MRI at the L4-L5 level (lumbarised S1) demonstrate an extruded intervertebral disc herniation with posterior migration, causing the significant compression of the thecal sac and displacement of adjacent nerve roots; the areas of interest are indicated by arrows in A3, B2, and B3. Contrast-enhanced sagittal (C1-C3) and axial (D1-D3) T1-weighted MRI at the same level show peripheral ring enhancement surrounding the extruded disc fragment, suggestive of inflammatory neovascularization and granulation tissue formation; arrows highlight the areas of interest in C2, D1, and D3, consistent with an ongoing resorptive process. A3, B2, B3 (T2-weighted images): arrows indicate the extruded disc fragment that has migrated posteriorly, causing the significant compression of the thecal sac and displacement of the adjacent nerve roots. C2, D1, D3 (post-contrast T1-weighted images): arrows highlight the peripheral ring enhancement surrounding the extruded disc fragment, representing vascularized granulation tissue and inflammatory changes consistent with disc resorption. MRI: magnetic resonance imaging

**Figure 3:**
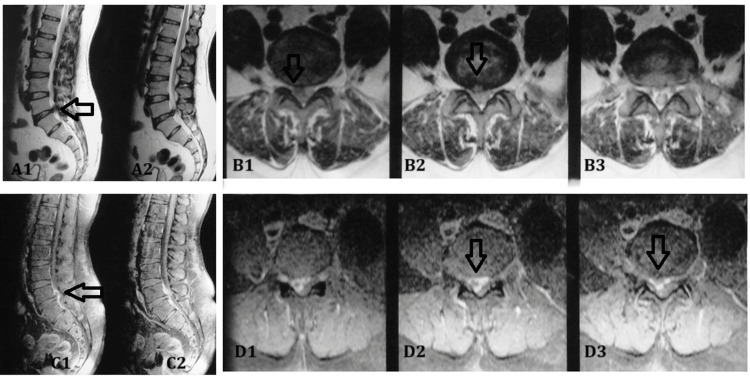
Sagittal (A1-A2) and axial (B1-B3) T2-weighted MRI at the L5-S1 level reveal a disc bulge resulting in the mild compression of the thecal sac; the areas of interest are marked by arrows in A1, B1, and B2. Corresponding contrast-enhanced sagittal (C1-C2) and axial (D1-D3) T1-weighted MRI do not demonstrate peripheral enhancement, suggesting an absence of active inflammatory or vascularized tissue response around the bulging disc; arrows highlight the areas of interest in C1, D2, and D3. A1, B1, B2 (T2-weighted images): arrows indicate the location of the disc bulge at L5-S1, associated with mild compression of the thecal sac. C1, D2, D3 (post-contrast T1-weighted images): arrows highlight the same disc region, where no peripheral enhancement is observed, supporting the absence of active inflammation or granulation tissue. MRI: magnetic resonance imaging

All patients, irrespective of the type of intervention, had a good clinical outcome at the end of the final follow-up. Multiple examples of rim enhancement are depicted in images (Figure 2, Figure 3).

## Discussion

Most intervertebral disc herniations resolve spontaneously (completely or partially), though the predictive course of disease remains obscure for the treating physician. In most cases, the natural course of a disc herniation involves its reduction in size over time [2]. Larger migrating type herniations are likely to regress more readily than smaller ones because of their tendency to penetrate the annulus fibrosus and posterior longitudinal ligament (PLL), thereby being exposed to systemic circulation in the epidural space. Historically, this phenomenon was first described in early literature by Teplick and Haskin [2], followed by clinical validation in prospective studies showing favorable outcomes with non-operative management [12,13]. Komori et al. further highlighted that extruded herniations were more prone to regression compared to contained protrusions [4]. A pivotal role for contrast-enhanced MRI in this setting was recognized by Bozzao et al. and later by Komori et al., who emphasized its value in tracking natural disc regression [5,6]. Most of the literature suggests a time frame of 6-8 weeks for the conservative trial of management, and the majority of the cases have symptomatic relief by then [2,3]. In cases with partial relief, there exists a dilemma for the continuation of conservative trial or any other modalities of treatment such as epidural steroids or surgery. Our study made an attempt to throw an insight into the possible ways of solving the dilemma to both the patient and treating surgeon.

Inflammation-mediated neovascularization has emerged as a key mechanism in spontaneous regression. Histopathological and immunohistochemical studies demonstrate that macrophage infiltration, particularly in extruded herniations, is closely associated with granulation tissue and neo-angiogenesis [14,15]. These macrophages secrete inflammatory cytokines, vascular endothelial growth factor (VEGF), and matrix-degrading enzymes that promote resorption. The direct association between VEGF-induced angiogenesis and disc regression was elucidated by Haro et al. [7], while Kato et al. [8] detailed the sequential dynamics of cytokine release. Arai et al. further confirmed macrophage-rich environments in herniated disc tissues [10]. Recent literature reinforces this mechanism. A meta-analysis by Zou et al. documented a pooled resorption rate of 70.4%, with sequestrated (87.8%) and extruded (66.9%) herniations showing higher spontaneous resolution than protrusions (37.5%) [14]. Zeng et al. noted that the "bull's-eye" rim sign on contrast-enhanced MRI predicted resorption with high specificity [15]. These findings are aligned with our observations that rim enhancement significantly correlates with clinical improvement. Yu et al. expanded on this understanding by showing that M2 macrophages, which express interleukin-10 (IL‑10) and transforming growth factor beta (TGF‑β), are central to the resorptive process, offering potential for future immunomodulatory therapies [16]. In our series, rim enhancement was observed in the majority of patients who improved with conservative therapy, with enhancement thickness ranging from 1 mm to 3.8 mm. Notably, Autio et al. reported that both the presence and thickness of rim enhancement were significant predictors of regression, an observation corroborated by our findings [12]. Findings of Komori et al. suggest that neovascularization fibrous tissue around a disc herniation, phagocytosis, and/or degradation is promoted and the herniated disc material resolves via these events [6].

All the above pathological events that occur are depicted radiologically on contrast-enhanced MRI alone [6]. Judging from above, the enhancement noted around the disc herniation represents vascularized fibrous tissue. Thickness of enhancement at baseline was the strongest determinant of the clinical resolution of symptoms in the previous study population, with higher enhancement thickness associated with better resolution of symptoms. Therefore, one can reasonably consider the intensity and extent of enhancement to represent the degree of inflammatory tissue reaction occurring around disc herniation. Our study had contrast thickness varying between 1 mm and 3.8 mm. In our study, there was a significant correlation of rim enhancement on contrast-enhanced MRI to clinical improvement of symptoms (p < 0.001). Patients who had no rim enhancement on contrast-enhanced MRI underwent surgery (n = 4) except for two patients who continued with conservative management and recovered over a period of six months.

The treatment protocol for patients of LDH presenting with acute lumbar radiculopathy is usually in the form of conservative management for a period of 4-6 weeks in the absence of any red flag signs. Failure of this treatment can be managed by injection methods before planning for surgical management. It is important to note that patients presenting with progressive worsening of neurological status with bowel or bladder involvement are not considered candidates for conservative management. These patients require urgent surgical decompression for optimum neurological recovery. On the contrary, patients with isolated back pain without any radiculopathy are not to be treated with the above protocol.

Advancements in imaging biomarkers are further refining prognostication. Hornung et al. developed a predictive model incorporating herniation volume, L4 vertebral body height, and sacral slope to anticipate resorption likelihood [17]. Case reports, such as that by Jeon et al., document complete resorption within nine months with concordant symptom resolution [18], while future directions include artificial intelligence models that integrate imaging data for individualized management.

Clinico-radiological correlation was observed in protrusion and extruded type of LDH; however, the sequestrated type of disc did not show any significant relation between rim enhancement and the reduction of follow-up VAS score in contrast to previous studies. Our study was the first to study the role of contrast-enhanced MRI in the management of LDH in the Indian population.

Our study has certain limitations. First, the relatively small sample size, particularly the very small number of patients in the non-enhancement group (n = 6), limits the statistical power and generalizability of the results. Second, although VAS scores provide a simple and widely used measure of pain severity, the absence of follow-up imaging prevented us from assessing longitudinal changes in enhancement patterns and their relationship to disc resorption. Third, the imbalance between enhancement and non-enhancement groups may have influenced comparative outcomes. These limitations should be considered when interpreting our findings.

## Conclusions

Contrast-enhanced MRI appears to be a valuable non-invasive tool in predicting the clinical course of LDH. The presence of rim enhancement around the herniated disc material was strongly associated with favorable outcomes following conservative treatment, while patients without rim enhancement were more likely to require surgical intervention. The thickness of rim enhancement and disc morphology, particularly extrusion and sequestration, also showed an association with symptomatic improvement, supporting the biological plausibility of an active resorption process. Nevertheless, these findings must be interpreted with caution due to the relatively small sample size, especially the limited number of patients in the non-enhancement group, which restricts the strength and generalizability of the results. Moreover, the absence of follow-up imaging precluded the direct assessment of radiological regression in relation to contrast enhancement. Despite these limitations, the prospective design and systematic clinico-radiological correlation remain important strengths, and larger multicenter studies with serial imaging and long-term follow-up are warranted to validate and expand upon these preliminary observations.
